# First record of the complete chloroplast genome of *Polygonatum infundiflorum* (Asparagaceae), a Korean endemic species

**DOI:** 10.1080/23802359.2023.2215349

**Published:** 2023-05-25

**Authors:** Se Ryeong Lee, Young-Ho Ha, Dong Chan Son, Sang-Chul Kim

**Affiliations:** Division of Forest Biodiversity, Korea National Arboretum, Pocheon, South Korea

**Keywords:** Asparagaceae, *Polygonatum infundiflorum*, endemic species, complete chloroplast genome

## Abstract

*Polygonatum infundiflorum* Y.S. Kim, B.U. Oh & C.G. Jang et al. 1998 is a Korean endemic species. This is first report on the complete chloroplast genome sequence of *P. infundiflorum*. The complete chloroplast genome length was 154,578 bp with a GC content of 37.7%. The large single-copy (LSC) region was 83,527 bp long, and the small single-copy (SSC) region was 18,457 bp long. The paired inverted repeats (IRs) were 26,297 bp and separated the LCS and SSC regions. There were 113 genes, comprising 78 protein-coding genes, four rRNA genes, 30 tRNA genes, and one pseudogene (*infA*). In total, 16 genes contained one intron, and two genes contained two introns. Phylogenetic analysis suggested that *Polygonatum* was divided into three sections, each forming a monophyletic group. *P. infundiflorum* was sister to *P. macropodum* and formed a monophyletic group with *P. inflatum*. This study provides basic information for future research and contributes to taxonomic and genetic studies on *Polygonatum*.

## Introduction

**Figure 1.**
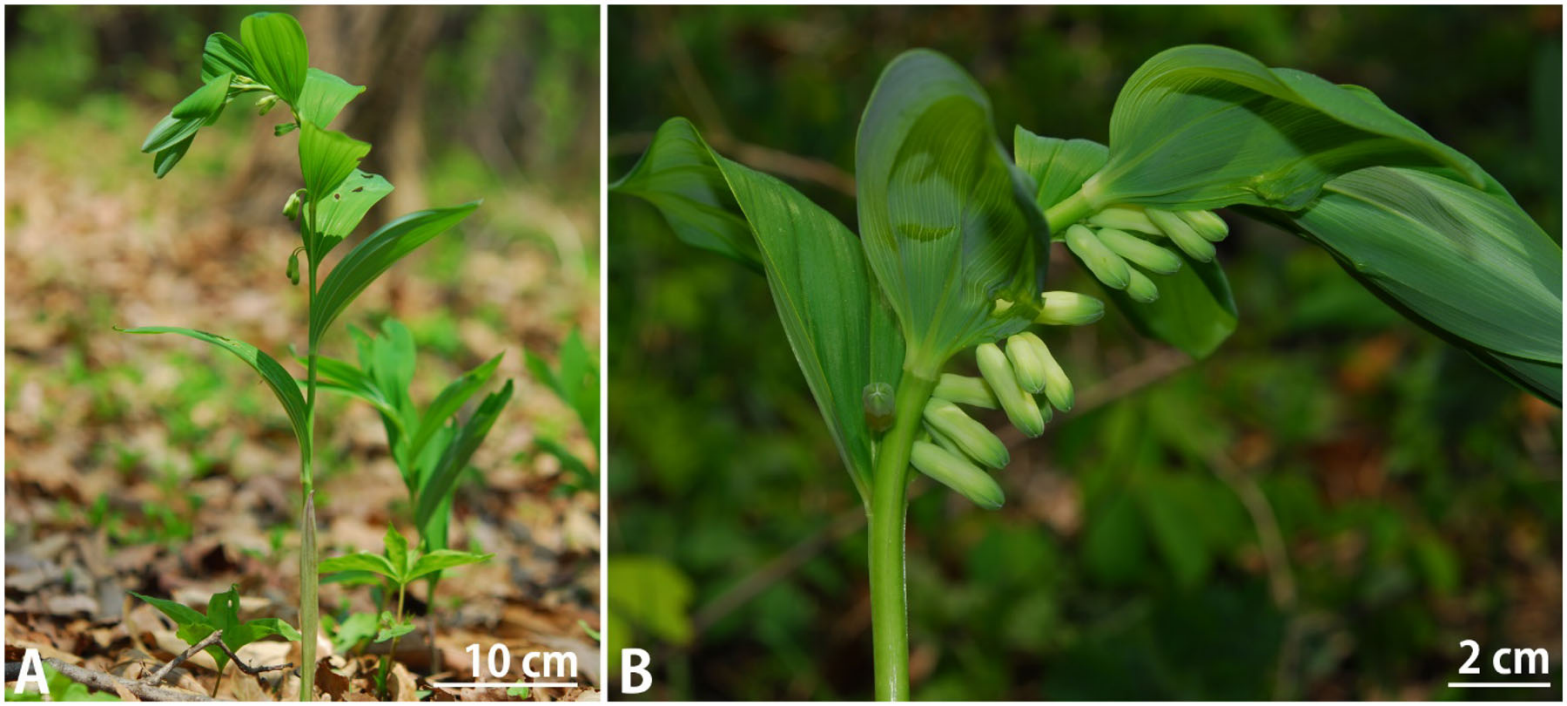
Photographs of *Polygonatum infundiflorum* (A) Habitat, (B) Inflorescence. Photo Credits: Sa-Bum Jang.

*Polygonatum* Mill. is belongs to Asparagaceae and is divided into three sections (*Polygonatum*, *Verticillata*, and *Sibirica*) based on phylogenetic studies (Meng et al. 2014; Floden and Schilling 2018). These species have been used in traditional medicine and are being actively studied in genetic systematics and marker development (Wujisguleng et al. 2012; Floden and Schilling 2018; Pan et al. 2020; Wang et al. 2022; Yang et al. 2022). *Polygonatum infundiflorum* Y.S. Kim, B.U. Oh & C.G. Jang et al. 1998 is a Korean endemic species discovered in Pungdo, Ansan-si, Gyeonggi-do (Jang et al. 1998; Oh et al. 2016). It belongs to the sect. *Polygonatum*, and can be distinguished from related species based on characteristics including curved stem tip, absence of trichomes along the midrib and margin on the abaxial leaf surface, erect stamens, and multicellular trichomes on filaments (Jang et al. 1998; Jang 2002; Figure 1). Although several studies have reported the genetics and complete chloroplast genomes of other *Polygonatum* species, few studies related to this species have been published. Therefore, we report the complete chloroplast genome of *P. infundiflorum* in this study to contribute to plant taxonomic identification, genetic diversity, and medicinal uses.

## Materials and methods

*P. infundiflorum* was collected from Daejeon, Korea (39°19′20.8ʺN, 127°28′57.1ʺE), and voucher specimens were stored at Korea National Arboretum herbarium (http://www.nature.go.kr/kbi/plant/smpl/KBI_2001_030100.do; received by Hee Young Gil, E-mail: warmishe@korea.kr) under the voucher number ESK21-311. Fresh leaves were dried using silica gel.

DNA was extracted using a DNeasy Plant Mini Kit (Qiagen Inc., Valencia, CA, USA) and DNA quality and concentration were confirmed through NanoDrop 2000 microspectrophotometer (Thermo Fisher Inc., Waltham, MA, USA). Next-generation sequencing (NGS) was generated for sequencing and data analysis using the TruSeq DNA PCR Free (550) library kit on the Illumina MiSeq platform (Macrogen, Inc., Seoul, Korea). The chloroplast sequence was assembled using GetOrganelle with ‘embplant_pt’ as a reference (Jin et al. 2020) and finally confirmed in Geneious Prime (Kearse et al. 2012). The coding sequence (CDS) and transfer RNA (tRNA) were annotated using GeSeq (Tillich et al. 2017) and tRNAscan-SE software (Lowe and Chan 2016). Genome maps, cis-splicing genes, and trans-splicing genes were generated using CPGview (Liu et al. 2023). The assembled sequence was registered in NCBI GenBank under the accession number OP764686.

We analyzed 30 *Polygonatum* species together with *Maianthemum*, *Convallaria*, and *Dracaena* of outgroups. We used the MAFFT program in PhyloSuite for 78 CDS sequences that were extracted, concatenated, and aligned (Zhang et al. 2020). We used the ModelFinder program in PhyloSuite (Kalyaanamoorthy et al. 2017) to find the best-fit models for the maximum likelihood (ML). The best-fit model of ML was TVM + F+R2 (AICc). The aligned sequence dataset was subjected to ML analysis using IQ-Tree with 10000 bootstrap replicates (Ronquist et al. 2012; Nguyen et al. 2015).

## Results and discussion

**Figure 2.**
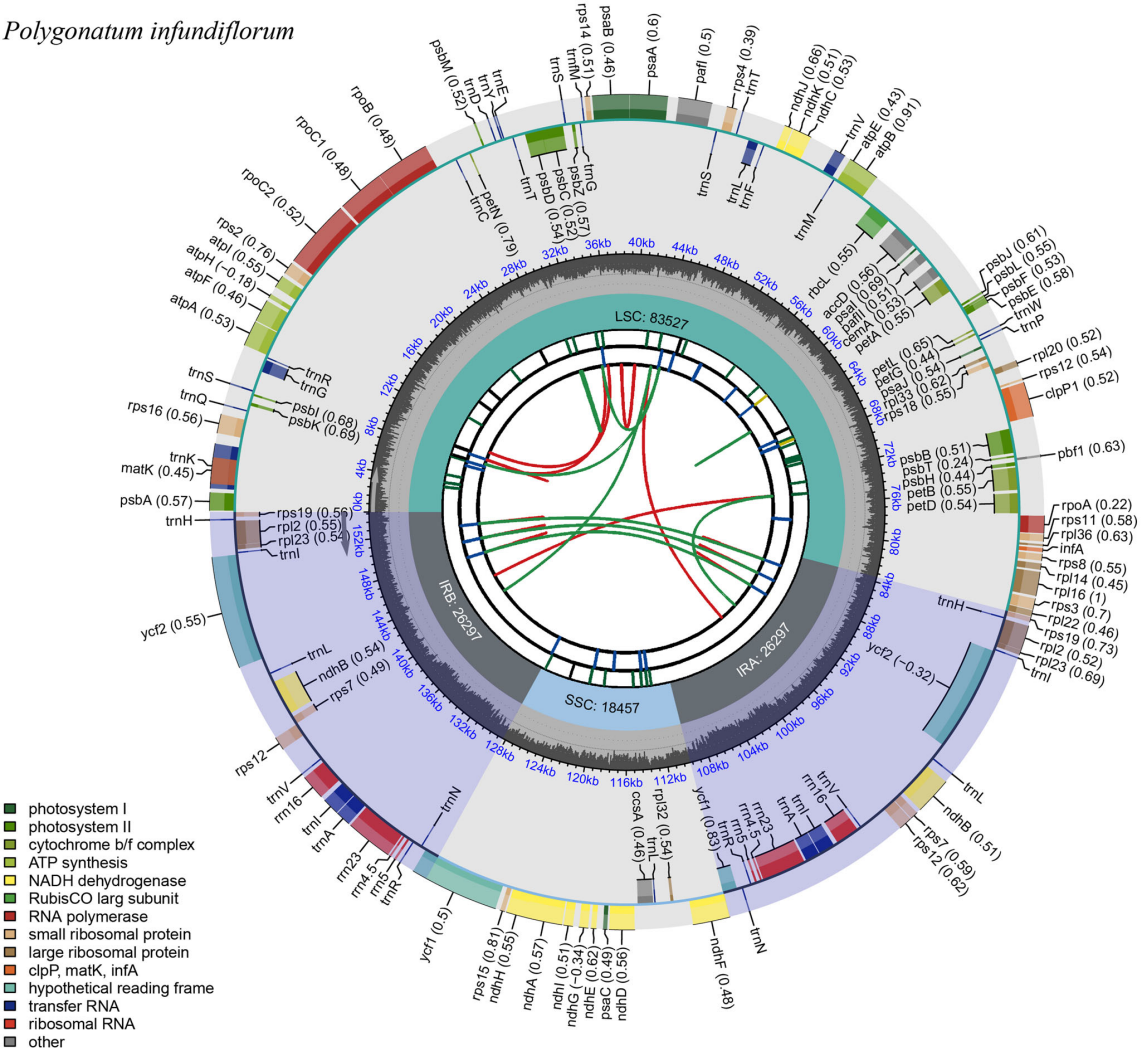
Complete chloroplast circle map of *Polygonatum infundiflorum* drawn using CPGview (Liu et al. 2023). The circle map contains six tracks. The first track indicates the forward and reverse directions in red and green, respectively. The second track is a blue bar representing tandem repeats. The third track represents microsatellite sequences in green and yellow. LSC, SSC, and inverted repeat (IRa and IRb) regions are shown on the fourth track. The fifth track represents GC contents along plasma, and the sixth track represents genes.

Of 7,914,172 reads, 33,856 reads were used to assemble the chloroplast genome of *P. infundiflorum* (maximum coverage: 115×, average coverage: 65.9×; Supplementary Figure 1). The sequence length of the complete chloroplast genome was 154,578 bp with a GC content of 37.7%. The length of the large single copy (LSC) region was 83,527 bp and that of the small single copy (SSC) region was 18,457 bp. The paired inverted repeats (IRs) were 26,297 bp, and separated the LCS and SSC regions (Figure 2). A total of 113 genes existed, comprising 78 protein-coding genes, four rRNA genes, 30 tRNA genes, and one pseudogene (*infA*). The *infA* gene has a premature stop codon and has also been identified in *Convallaria* and *Dracaena* within the Asparagaceae family (Zhu et al. 2018; Raman et al. 2021). In total, 16 genes (10 protein-coding genes: *atpF, ndhA, ndhB, petB, petD, rpl2, rpl16, rpoC1, rps12,* and *rps16*; six tRNA: *trnA-UGC, trnG-UCC, trnl-GAU, trnK-UUU, trnL-UAA,* and t*rnV-UAC*) contained one intron and two genes (*clpP1* and *pafI*) contained two introns. The *rps12* is a trans-splicing gene (Supplementary Figure 2B) and thirteen genes including *rps16, atpF, rpoC1, pafI, clpP1, petB, petD, rpl16, rpl2, ndhB, ndhA, ndhB,* and *rpl2* are cis-splicing genes (Supplementary Figure 2A).

**Figure 3.**
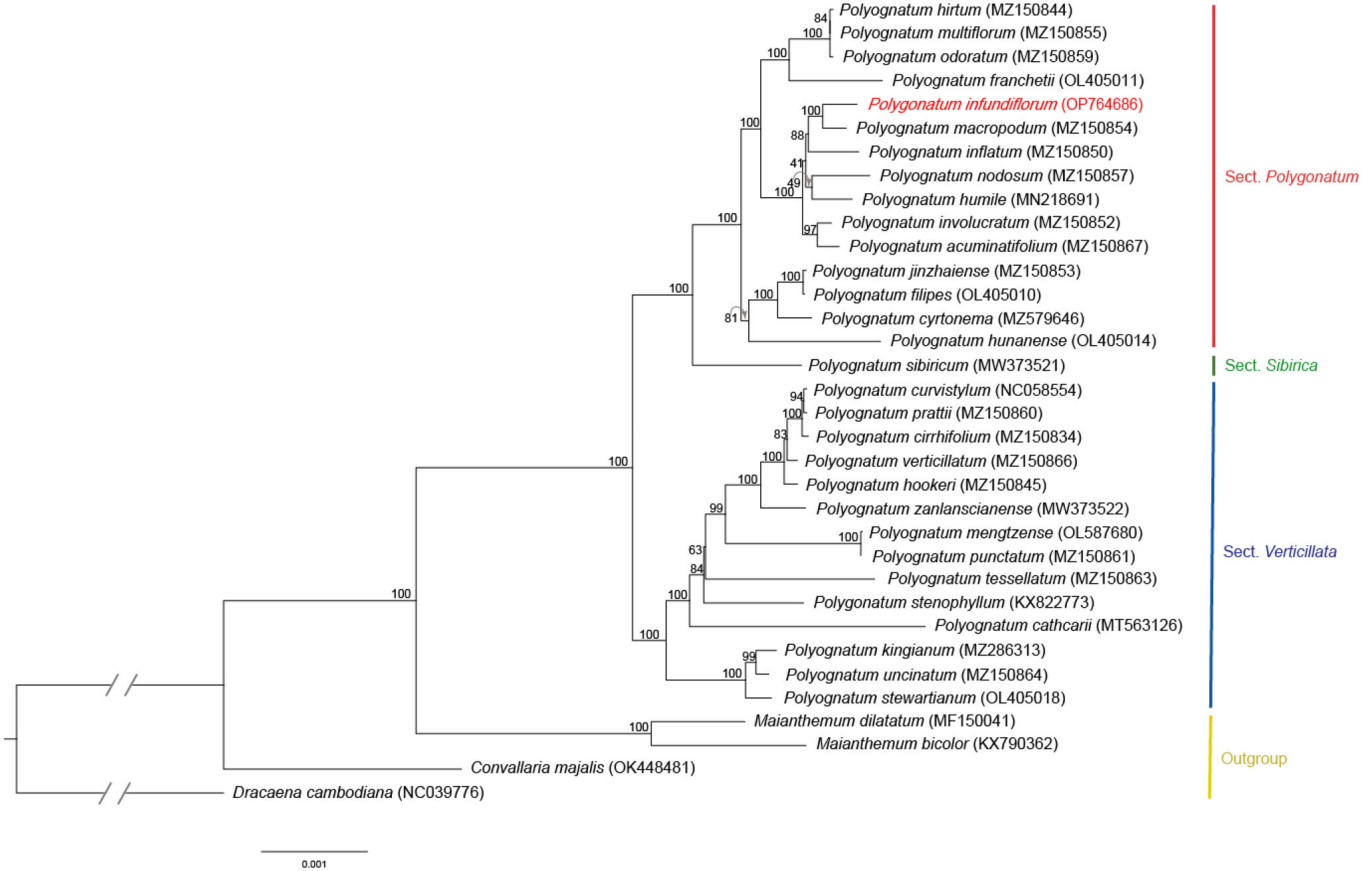
Maximum likelihood (ML) tree of 34 chloroplast genomes of *Polygonatum* and the outgroups. Values indicated above the branches are ML bootstrap support values (bootstrap repeat: 10000).

The ML tree for the 78 CDS sequences were analyzed including 30 *Polygonatum* species and four outgroup species (*Maianthemum*, *Convallaria*, and *Dracaena*). The concatenated CDS length was 64476 bp. In the phylogenetic tree, outgroups branched separately, and *Polygonatum* was divided into three sections, each forming a monophyletic group. *P. infundiflorum* was sister to *P. macropodum* and formed a monophyletic group with *P. inflatum*. This clade indicated good support (BS = 100% and 88%, respectively), suggesting a close phylogenetic relationship between these three species (Figure 3).

This study provides the cp genome information of *P. infundiflorum*, which would contribute to the taxonomic and genetic studies on *Polygonatum*.

## Supplementary Material

Supplemental Material

Supplemental Material

Supplemental Material

## Data Availability

The genome data that support the findings of this study are available at NCBI (https://www.ncbi.nlm.nih.gov/) under the accession number OP764686. The associated BioProject, SRA, and Bio-Sample numbers are PRJNA897089, SRR22184768, and SAMN31577749, respectively.
