# Supplementary figures and images for: First record of the complete chloroplast genome of Polygonatum infundiflorum (Asparagaceae), a Korean endemic species
Source: Mitochondrial DNA B Resour. 2023 May 25;8(5):603–6. doi: 10.1080/23802359.2023.2215349 (PMC10215011; doi:10.1080/23802359.2023.2215349)

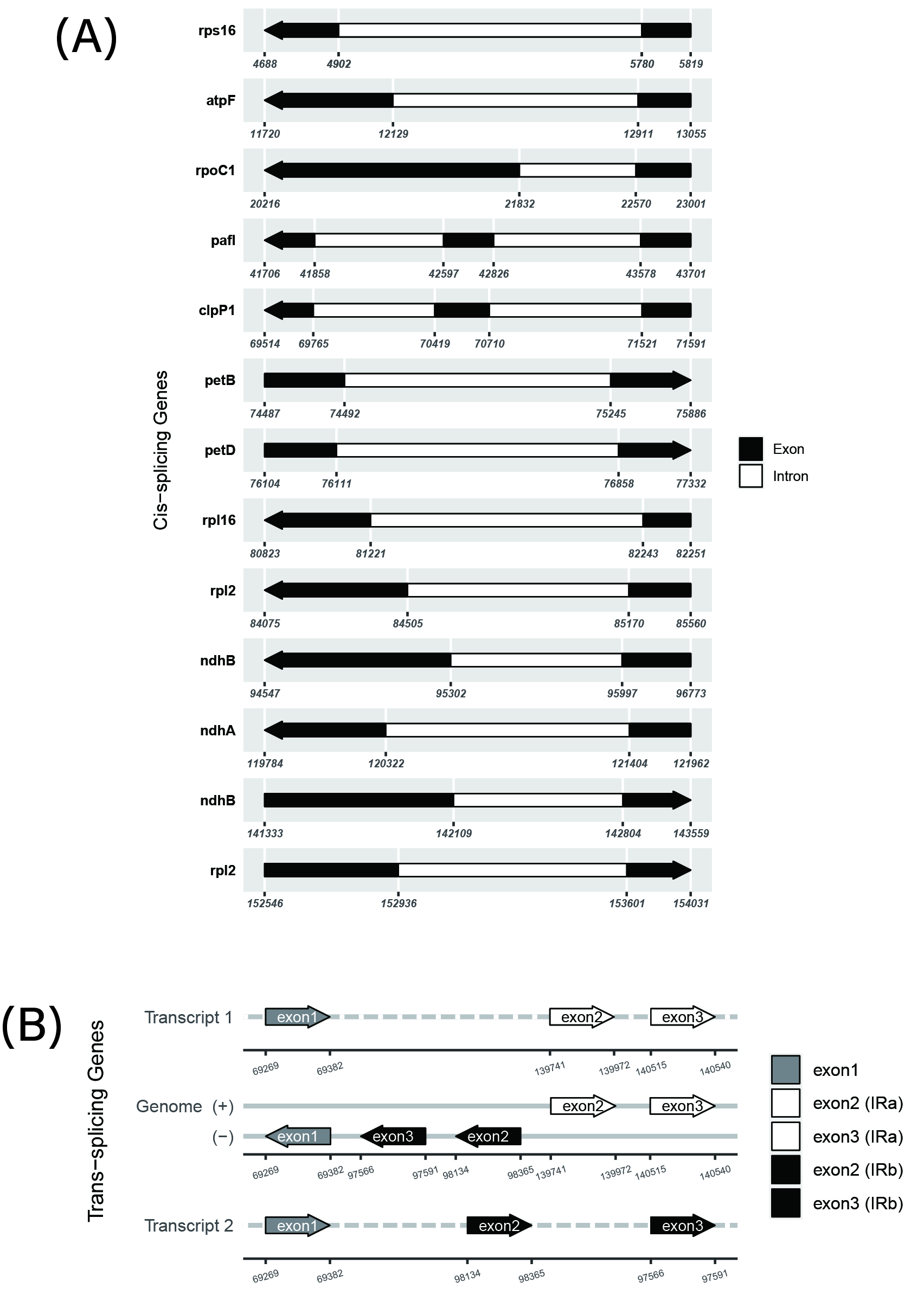

Supplement: Supplemental Material [file TMDN_A_2215349_SM1131.jpg]

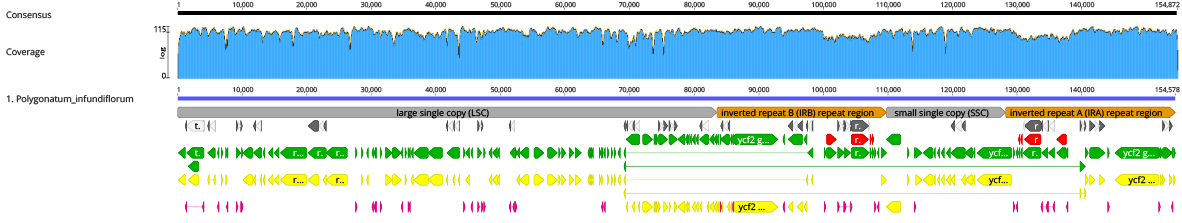

Supplement: Supplemental Material [file TMDN_A_2215349_SM1127.jpg]
