# Supplementary material for: First record of the complete chloroplast genome of Polygonatum infundiflorum (Asparagaceae), a Korean endemic species
Source: Mitochondrial DNA B Resour. 2023 May 25;8(5):603–6. doi: 10.1080/23802359.2023.2215349 (PMC10215011; doi:10.1080/23802359.2023.2215349)
Supplement: Supplemental Material [file TMDN_A_2215349_SM1124.docx]

**Supplemental material**

Supplementary Figure 1. Overall coverage depth of the chloroplast genome assembly of *Polygonatum infundiflorum*

Supplementary Figure 2. Schematic of the cis-splicing gene and trans-splicing genes maps generated for the chloroplast genome of *Polyognatum infundiflorum*


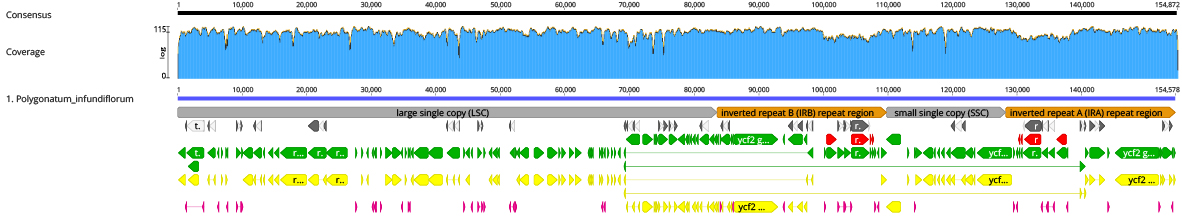


Supplementary Figure 1. Overall coverage depth of the Chloroplast genome assembly of *Polygonatum infundiflorum*. This figure was generated using Geneious Prime by aligning DNA-Seq data to the whole chloroplast genome. The height of the blue graph indicates the number of sequences at each location.


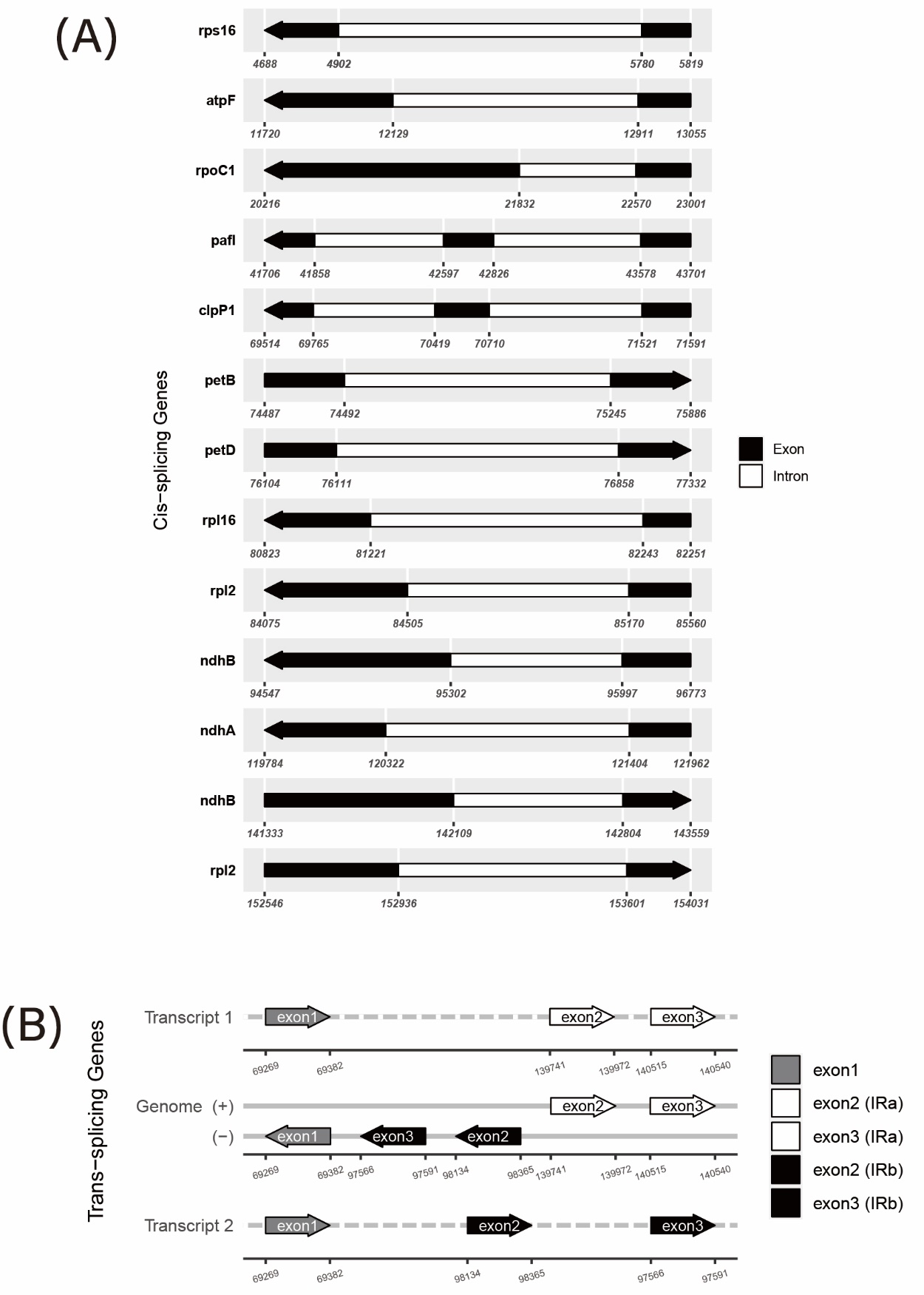


Supplementary Figure 2. Schematic of the (A) cis-splicing gene and (B) *rps12* trans-splicing genes maps generated for the Chloroplast genome of *Polyognatum infundiflorum.* The map was generated using CPGview and black and white indicate exon and intron, respectievely.
